# The antitumor activity and preliminary modeling on the potential mechanism of action of human peroxiredoxin-5

**DOI:** 10.18632/oncotarget.16089

**Published:** 2017-03-10

**Authors:** Juanjuan Liu, Xiaozhou Feng, Yuanyuan Jin, Zhengyang Sun, Haoyi Meng, Zhifei Zhang, Laixing Hu, Zhaoyong Yang

**Affiliations:** ^1^ Institute of Medicinal Biotechnology, Chinese Academy of Medical Sciences and Peking Union Medical College, Beijing, People's Republic of China; ^2^ College of Pharmaceutical Sciences, North China University of Science and Technology, Tangshan, People's Republic of China

**Keywords:** anti-cancer bioactive peptide, human peroxiredoxin-5, antitumor activity, immunoregulation, PD-L1

## Abstract

Goat peroxiredoxin-5 (gPRDX5) was verified as a good anti-cancer bioactive peptide (ACBP) against different tumor cell lines. Considering the immunogenicity between species for further therapeutic application, it is necessary to similarly investigate the antitumor activity of human peroxiredoxin-5 (hPRDX5) with 89% similarity in sequence to gPRDX5. In order to evaluate its antitumor activity, the potential anti-neoplastic effect of hPRDX5 on a mouse model was observed directly. The results of its *in vivo* antitumor activity suggested that hPRDX5 could resist immunosuppression by promoting lymphocyte proliferation and up-regulating the levels of serum cytokines. Meanwhile, PD-L1 was speculated as one of the targets of hPRDX5 to inhibit tumor by enhancing the immune activity according to a preliminary molecular docking study on the interactions between hPRDX5 and PD-L1. The modeling provides a basis for structural modification on hPRDX5/PD-L1 for further biological and biochemical study on the pathway blocking mechanism of hPRDX5. In this work, the results demonstrate that hPRDX5 displays efficient antitumor and immunoregulatory properties in the colon cancer C26/BALB/c and melanoma B16/C57Bl/6 mice tumor models, and suggest the potential of developing peptides from hPRDX5 as low molecular weight drug candidates for corresponding cancer immunotherapy.

## INTRODUCTION

The goat peroxiredoxin-5 (gPRDX5) is an anti-cancer bioactive peptide (ACBP), which was first identified by Xiulan Su [1] from goat spleen or liver which was immunized with human gastric cancer protein extract. In long-term animal experiments, this ACBP exhibited potential antitumor activities without measurable side effects [1–4]. And, its anti-cancer effect was mainly exerted by affecting cell cycle and inducing cell apoptosis [5]. In combination with Cisplatin, gPRDX5 also reduced chemotherapy dosage and decreased toxicity, thus improving the life quality of xenograft nude mice bearing human gastric cancer [5–8]. However, the procedure of immunization and purification to acquire the peptide was complicated and time-consuming, and the yield was low as well. Therefore, we identified the amino-acid sequence of the peptide by employing 2D-nano-LC-ESI-LTQ-Orbitrap MS/MS in combination with Mascot database search in the goat subset of the Uniprot database and purified the corresponding protein by heterogeneous expression [9]. Thereafter, the anti-cancer bioactivity of gPRDX5 was confirmed with several kinds of tumor-cells and indicated that it was a good anti-cancer candidate, especially against B16 cells.

Considering the immunogenicity between species for further therapeutic applications, the human homologue protein of gPRDX5 was designed for further investigation on its antitumor activity, based on the speculation that homologous proteins in sequence have similar structures and thus similar functions. Therefore, the sequence of gPRDX5 was analyzed by BLAST on the website http://blast.ncbi.nlm.nih.gov/Blast.cgi to search its human homologue, and the result showed that the human peroxiredoxin-5 (hPRDX5) was 89% similar to gPRDX5.

The peroxiredoxins (PRDXs) are a ubiquitous family of antioxidant enzymes which catalyze the reduction of hydrogen peroxide, alkyl hydroperoxides and peroxynitrite [10]. Mammalian cells express six isoforms of PRDXs (PRDX1 to PRDX6), and all these six isotypes have been discovered in distinct subcellular locations and show a wide tissue distribution [11]. Peroxiredoxin 5 (PRDX5), the last isoform identified in the peroxiredoxin family, possesses unique properties [12]. First, PRDX5 is the only mammalian member of the atypical 2-Cysteine (2-Cys) PRDX subfamily [13]. Second, PRDX5 exhibits a remarkably wide subcellular localization such as mitochondria, cytosol, peroxisomes, and nucleus [14]. And third, PRDX5 plays many vital roles under physiopathological conditions. For instance, overexpression of PRDX5 in human tendon cells induces apoptosis following H_2_O_2_ treatment [15], up-regulation of PRDX5 has been reported in osteoarthritic cartilage and in TNF-α or IL-1β treated cartilage explants from patients with osteoarthritis [16], and PRDX5 was proven to be an anti-fibrotic effector that sustains renal physiology by inhibiting stat3 activation in rat kidney interstitial fibroblast cells [17]. However, the antitumor effect of PRDX5 has not yet been reported substantially. In view of this, we investigate the antitumor potential of hPRDX5, triggered by the antitumor activities of gPRDX5, herein in details.

An intact immune system is capable of recognizing and eliminating tumor cells through immune checkpoints. However, tumors can adapt to and circumvent these natural defense mechanisms [18–20]. The interaction between the programmed death 1 (PD-1) receptor and its ligand 1 (PD-L1) is a key pathway hijacked by tumors to suppress immune control [21–24]. Programmed cell death-1 (PD-1), an immunoinhibitory receptor of the CD28 family, which plays a major role in tumor immune escape [25, 26], is an inhibitory receptor expressed on the surface of T cells that physiologically limits T-cell activation and proliferation [27]. PD-L1 is one of two PD-1 ligands and is expressed on both antigen-presenting cells and T cells. Binding of PD-L1 to the PD-1 receptor regulates T cells negatively, causing decreased production of the effector cytokines, such as IL-2 and IFN-γ [28–30]. Therefore, blocking these interactions showed outstanding promise in restoring T cells' activity and reactivating the immune system to recognize and eradicate tumor and infected cells [31–33].

In our investigation, by taking advantage of molecular docking, the PD-L1 was selected as the potential target of hPRDX5 in the preliminary modeling for two reasons. First, PD-L1 is highly up-regulated in many types of tumor cells, such as melanoma, ovarian and lung cancers [34]. Second, the preliminary mechanism study revealed that hPRDX5 could increase the content of IL-2 significantly, which may function by inhibiting PD-L1. The potential mechanism by which the binding of hPRDX5 to PD-L1 exerts antitumor activity, by blocking the pathway of PD-1/PD-L1, was discussed based on a predicted structural modeling of hPRDX5-PD-L1 complex for further biological investigations.

## RESULTS AND DISCUSSION

### Antitumor activity in C26-injected mice

Colorectal cancer (CRC) is the third most common cancer worldwide and the fifth leading cause of death related to cancer in China [35]. Therefore, we monitored the tumor growth in a mouse model of colon cancer. We found significant decreases in the tumor growth in mice treated with hPRDX5 in a dose-dependent manner. The inhibition rate of 150 mg/kg hPRDX5 was 32.16%, which demonstrated that hPRDX5 inhibited tumor growth in C26-injected mice (Figure 2). In order to preliminarily explore the action mechanism of hPRDX5, the effects of hPRDX5 on spleen and thymus indices of C26-injected mice were evaluated. Both of them were increased significantly compared with control group when the mice were treated with hPRDX5 in a dose-dependent manner (Figure 3), indicating that hPRDX5 was able to counteract the effect of immunosuppression on immune organs development and protect the immune organs against the impairment caused by tumor. Hence, we hypothesize that it possesses the capacity of suppressing tumor through regulating organism immune function.

**Figure 1:**
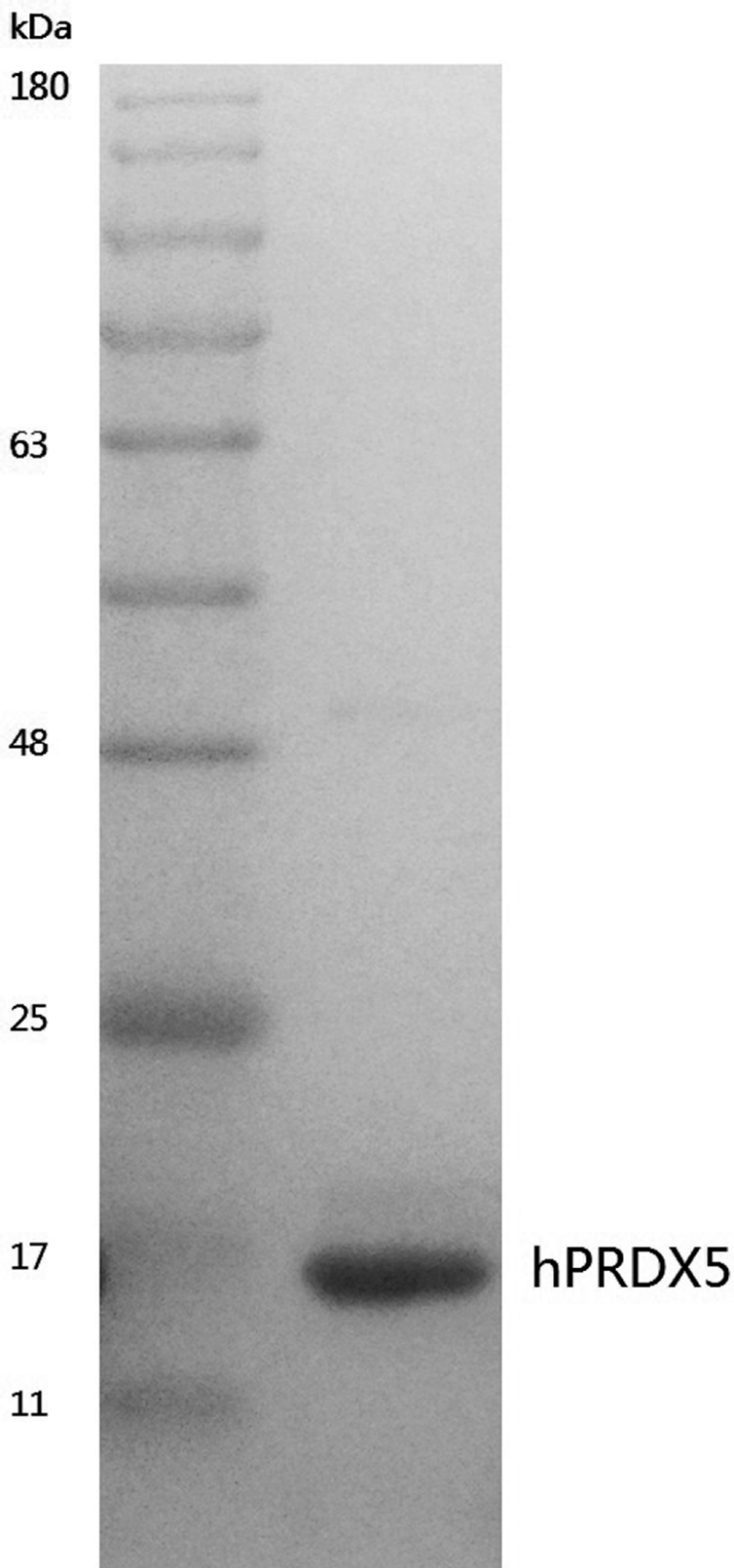
Coomassie stained SDS-PAGE gel of purified hPRDX5 The DNA fragment of hPRDX5 was constructed to expression vector pET-28a(+) and the plasmid was then transformed to BL21 (DE3) for heterogeneous expression. The hPRDX5 was purified by Affinity Chromatography and the molecular weight was about 17 kDa.

**Figure 2:**
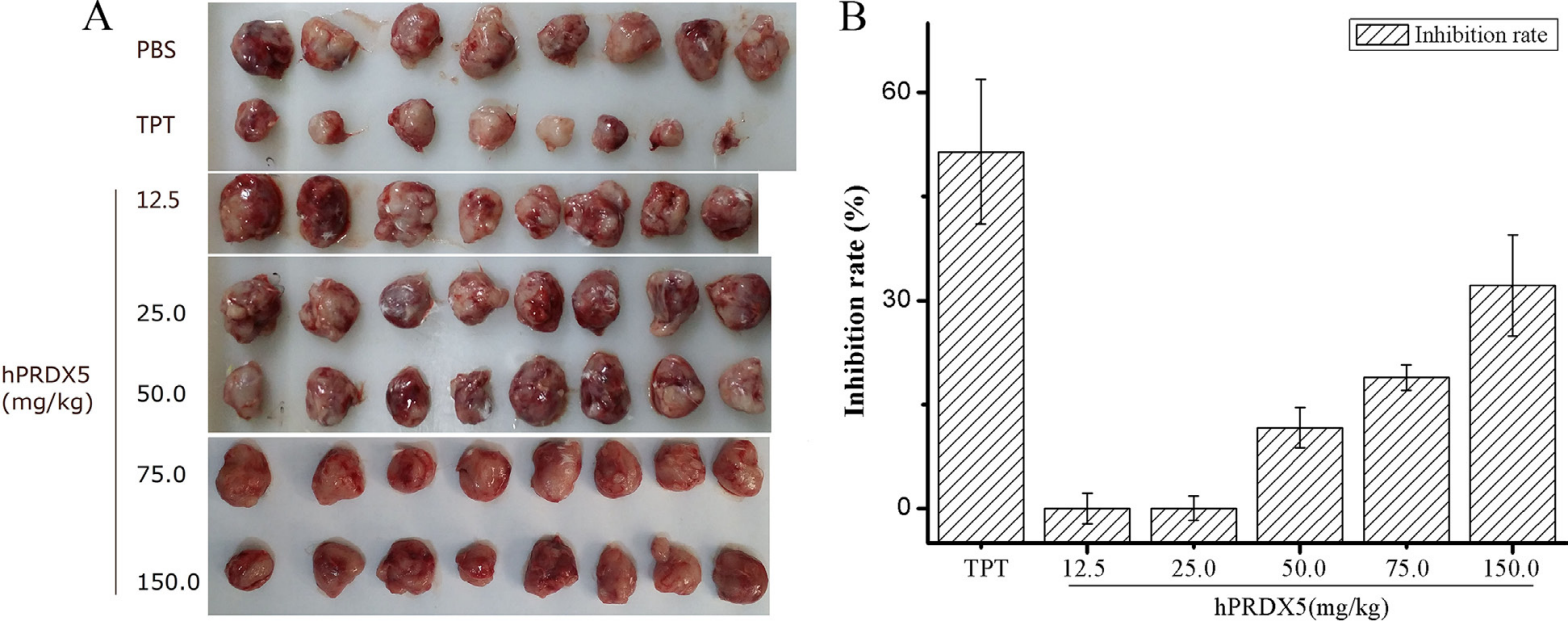
The anti-cancer bioactivity of hPRDX5 in C26-injected mice (**A**) Tumors were harvested after treated with hPRDX5 and controls (TPT and PBS). (**B**) The inhibition rate of tumor was evaluated by measuring the tumor weight compared with solvent control. The tumor growth was decreased significantly in mice treated with hPRDX5 in a dose-dependent manner. The highest inhibition rate was 32.16 %, which demonstrated that hPRDX5 was likely to a potential tumor suppressor. (*P* < 0.05 compared to control group).

**Figure 3:**
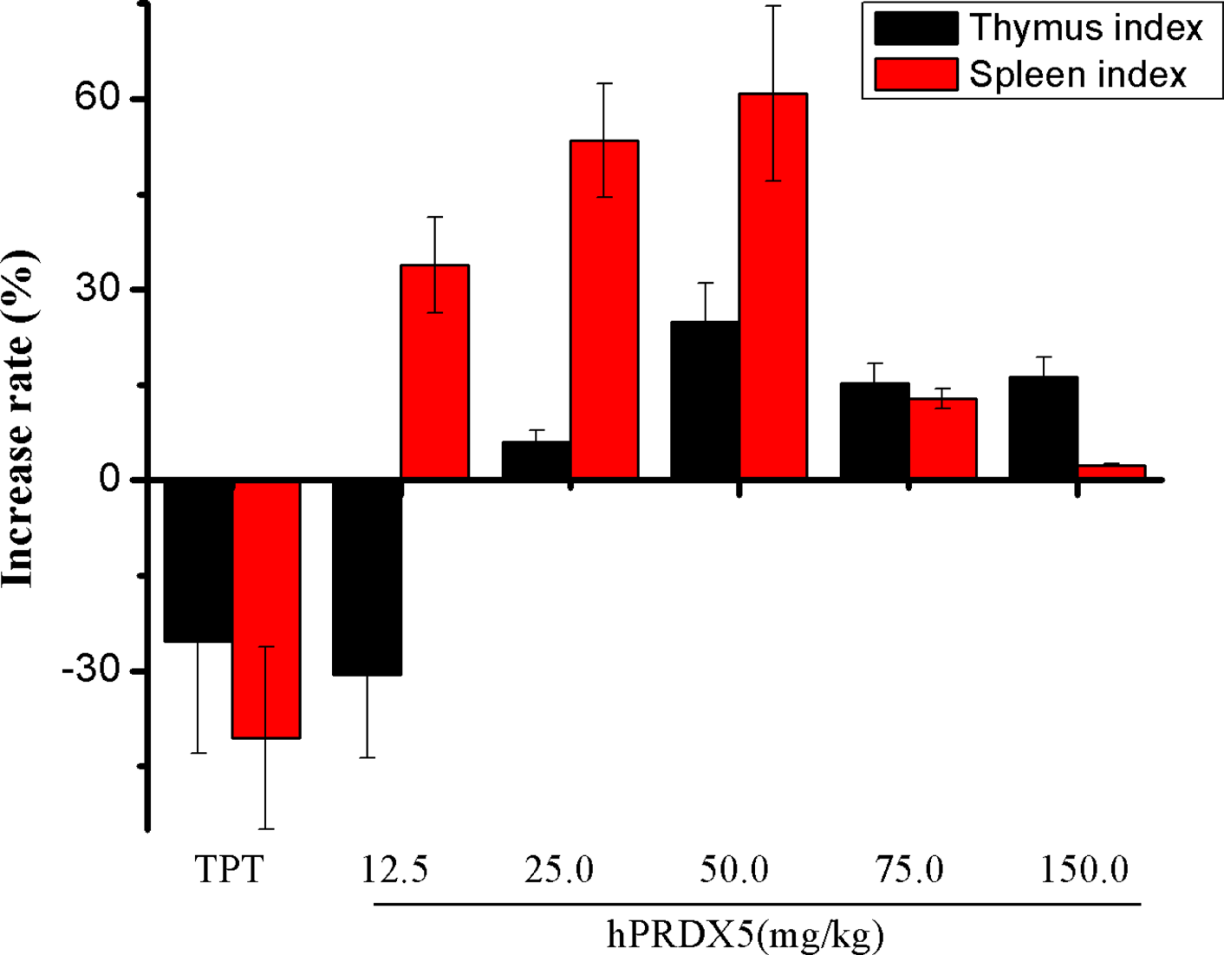
Effects of hPRDX5 on spleen and thymus indices in C26-injected mice The spleen and thymus indices were increased significantly compared with control group when the mice were given intraperitoneal injection of hPRDX5 at 12.5, 25.0, 50.0, 75.0 or 150.0 mg/kg, respectively (*P* < 0.05 compared to control group).

### Antitumor activity in melanoma B16-bearing mice

To get insight into its activity in tumor immunity and function mechanism, we observed the tumor growth in melanoma B16-bearing mice, which is commonly used to investigate tumor immunity. We found significant decreases in the tumor growth in mice treated with 75.0 mg/kg hPRDX5, which is the optimal dose in preliminary experiments, compared with those observed in mice treated with IL-2 (25.0 ng/kg), IFN-γ (5.0 μg/kg) or PBS. The results demonstrated that the inhibition rate of hPRDX5 was above 39% (Table 1), which suggested that hPRDX5 was likely to possess broad-spectrum anti-tumor activity *in vivo*. To identify the potential underlying mechanism of tumor suppression of hPRDX5, its effects on the proliferation of mitogen-induced splenic lymphocytes and the levels of cytokines were evaluated subsequently.

**Table 1:** The anti-cancer bioactivity of hPRDX5 in a melanoma B16/C57Bl/6 mice tumor model

Group	Dose	Number of animals	Tumor weight (g)	Inhibition rate (%)
Solvent control		10/10	2.70 ± 0.26	
IL-2	25.0 ng/kg	8/8	1.77 ± 0.57***	34.47
IFN-γ	5.0 μg/kg	8/8	2.09 ± 0.59*	22.71
hPRDX5	75.0 mg/kg	8/8	1.63 ± 0.36***	39.44

The inhibition rate of 75 mg/kg hPRDX5 was above 39 %, whereas the inhibition rates of IL-2 and IFN-γ were about 34% and 22%, respectively (*P < 0.05, ***P < 0.001 compared to control group). Data are means ± SD of eight animals.

**Figure 4:**
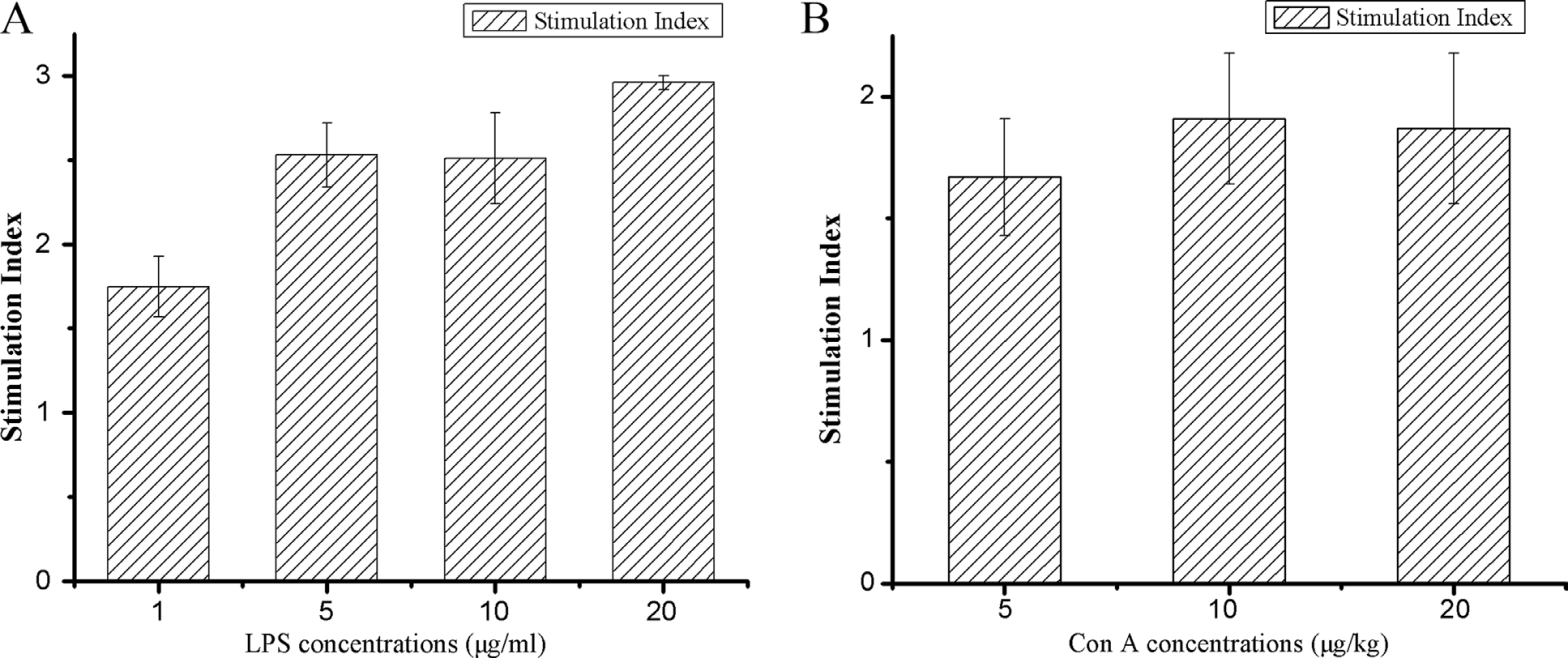
Effect of hPRDX5 on mitogen-induced splenic lymphocyte proliferation (**A**) LPS; (**B**) ConA. The proliferative responses of splenic lymphocytes to concanavalin A and LPS were enhanced significantly as compared with the control group. The proliferation of lymphocytes were enhanced by 75.0 mg/kg hPRDX5 with the stimulation index of 1.75, 2.53, 2.51 and 2.96 in the presence of LPS at 1, 5, 10, 20 μg/ml, respectively. Likewise, in the presence of concanavalin A at 5, 10, 20 μg/ml hPRDX5 elicited an increase in lymphocytes proliferation by 1.67, 1.91 and 1.87, respectively. (*P* < 0.05 compared to control group)

Lymphocytes are the key effector cells of mammalian adaptive immune system. Lymphocyte proliferation is the direct indicator reflecting the state of immunity in animal. Cell-mediated immunity can exert the actions of anti-infection, antitumor, helping lymphocyte produce antibody by sensitized lymphocyte against corresponding antigen [36]. In contrast, humoral immunity refers to antibody production and the accessory processes that accompany it, including: Th2 activation, germinal center formation and isotype switching, affinity maturation and memory cell generation. As generally known, concanavalin A stimulates lymphocyte T cells, which are involved in cell-mediated immunity, and LPS stimulates B cell proliferation are responsible for humoral immune response. Therefore, we investigated the effect of hPRDX5 on lymphocyte proliferation with mitogen stimulation. As illustrated in Figure 3, the proliferative responses of splenic lymphocytes to concanavalin A and LPS were enhanced significantly compared with the control group. The proliferation of lymphocytes were enhanced by hPRDX5 with the stimulation index of 1.75, 2.53, 2.51 and 2.96 in the presence of LPS at 1, 5, 10, 20 μg/ml, respectively (Figure 4A). Likewise, in the presence of concanavalin A at 5, 10, 20 μg/ml hPRDX5 elicited an increase in lymphocytes proliferation by 1.67, 1.91 and 1.87, respectively (Figure 4B). These results suggested that hPRDX5 improved both cellular and humoral immunity in melanoma B16-bearing mice by enhancing both T cell and B cell proliferation.

**Figure 5:**
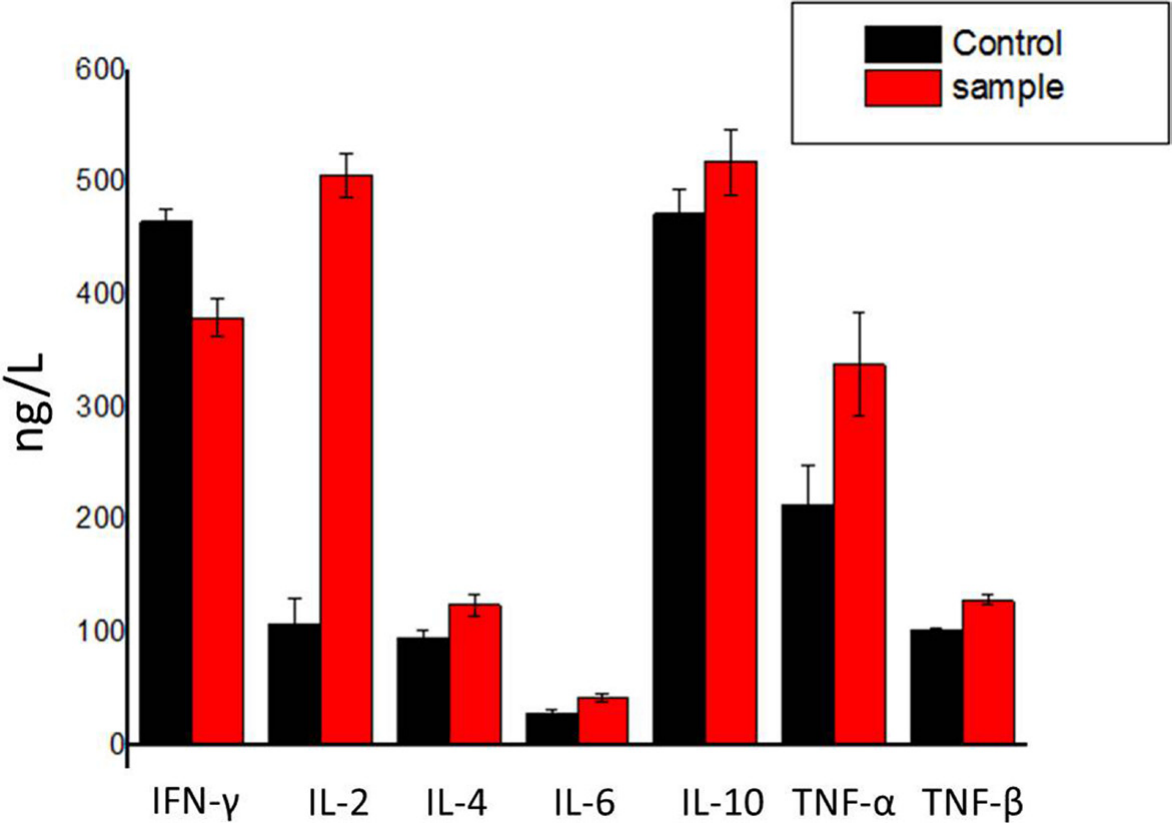
Effect of hPRDX5 on the levels of cytokines The levels of IL-2, IL-4, IL-6, IL-10, TNF-α and TNF-β were increased by 75.0 mg/kg hPRDX5, but IFN-γ was reduced slightly. The secretion of IL-2 was promoted most significantly. Data are means ± SD of eight animals (*P* < 0.05 compared to control group).

Cytokines play an important role in cell-cell communication in the immune system. Thereupon, we determined the effects of hPRDX5 on the levels of cytokines. The levels of interleukin-2 (IL-2), interleukin-4 (IL-4), interleukin-6 (IL-6), interleukin-10 (IL-10), tumor necrosis factor-α (TNF-α) and tumor necrosis factor-β (TNF-β) increased significantly except interferon-γ (IFN-γ) reduced slightly (Figure 5), which indicated that hPRDX5 could promote the secretion of IL-2, IL-4, IL-6, IL-10, TNF-α and TNF-β. Therefore, we speculate that hPRDX5 has regulatory effects on inflammation and lymphocyte functions through these cytokines secretion to possess its anti-cancer activities.

### Interaction between hPRDX5 and PD-L1 through molecular modeling

**Figure 6:**
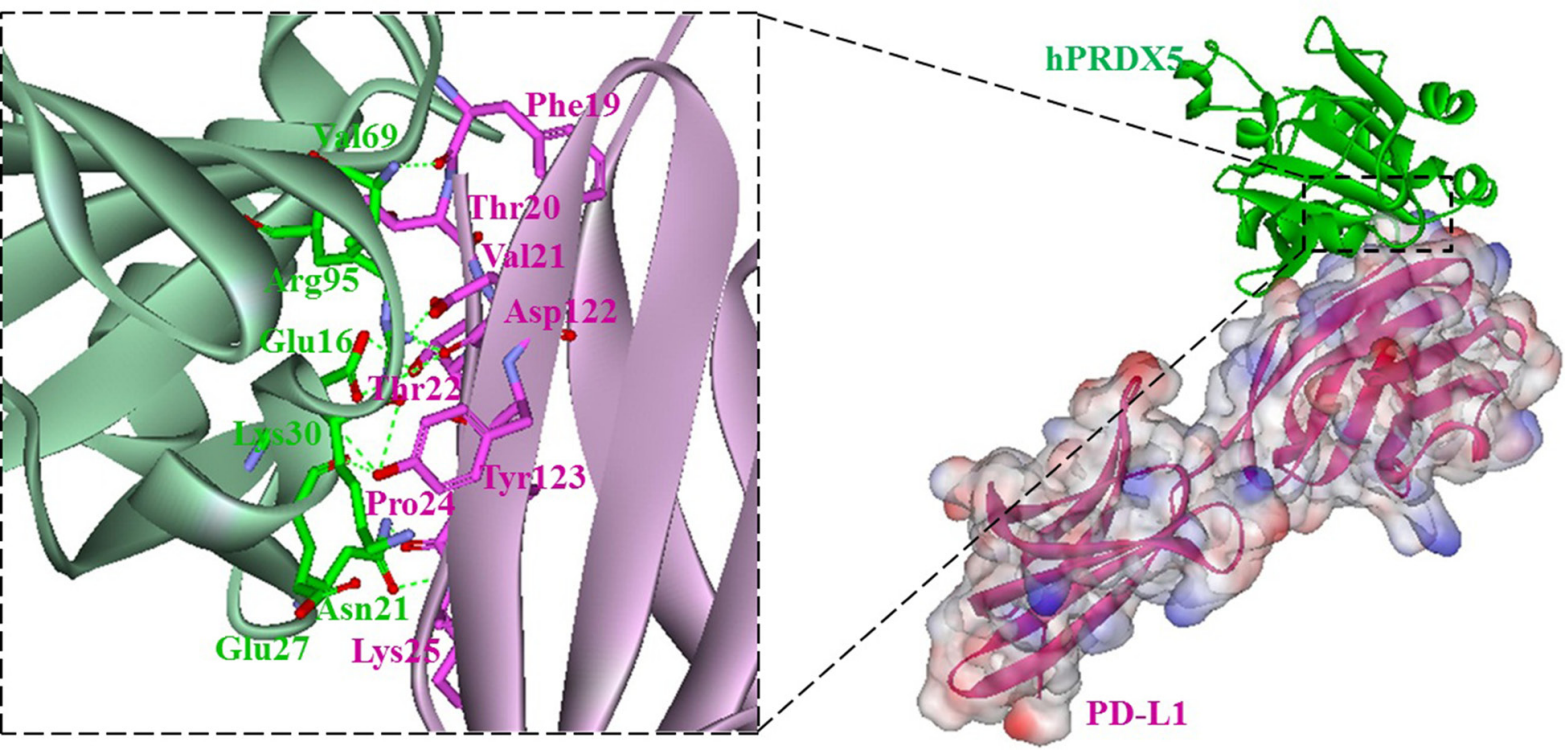
Overall structure of the hPRDX5/PD-L1 complex (right) hPRDX5 and PD-L1 are shown in green (ribbon diagram) and pink (surface representation), respectively; Close-up views of interfaces. Residues involved in hydrogen bonds (green dashes) are shown.

**Figure 7:**
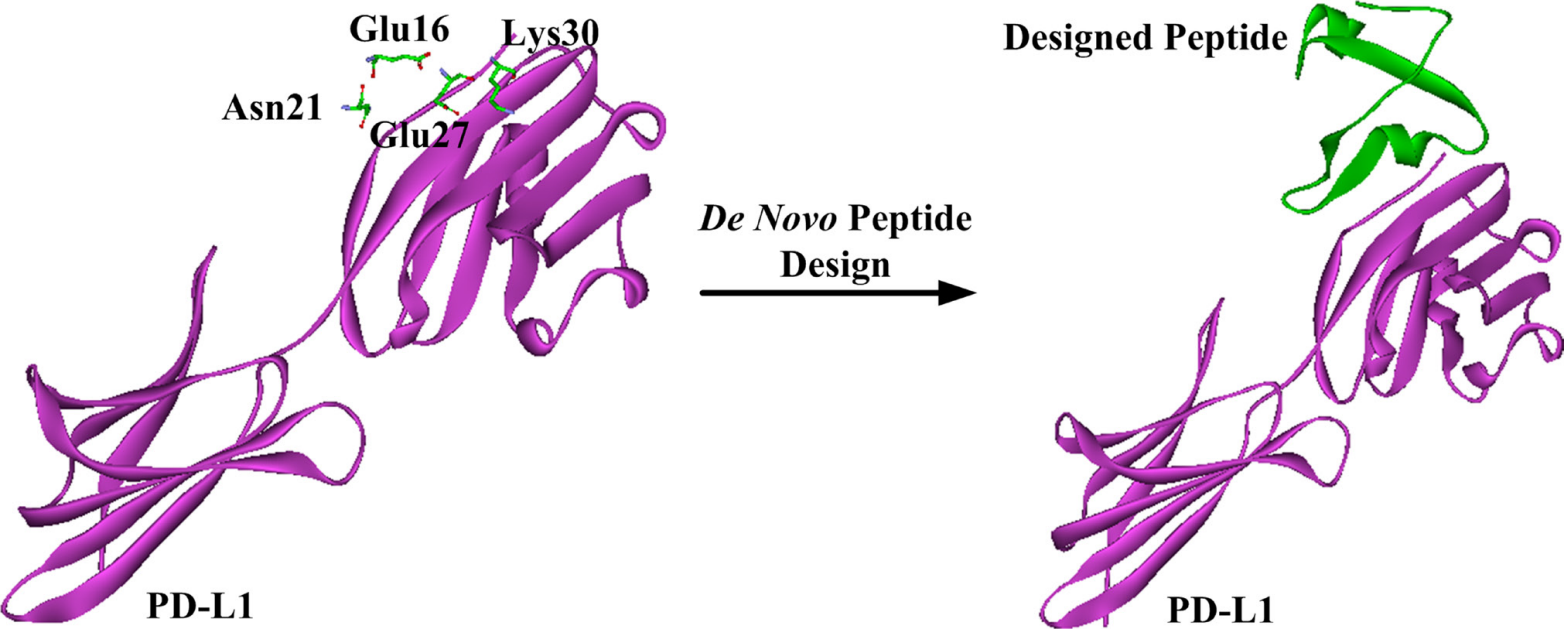
Schematic representation of *de novo* peptide design

Based on the previously proposed sites of PD-L1 residues important for the inhibitor binding [37, 38], we carried out a modeling study to investigate the potential interaction between hPRDX5 and PD-L1. In the predicted model, almost all the key interactions between hPRDX5 and the binding site of PD-L1 are conducted by the four conservative residues (Glu16, Asn21, Glu27 and Lys30) in hPRDX5 (Figure 6): For example, there are three hydrogen bonds between Lys30 of hPRDX5 and Tyr123 of PD-L1, Asn21 of hPRDX5 and Pro24 of PD-L1, and Arg95 of hPRDX5 and Asp122 of PD-L1. The conservative nature of these residues in hPRDX5 and other PRDX enzymes suggests that their important roles in forming the binding interface with PD-L1. Based on the current modeling results, the *in vivo* investigation on the potential interaction between hPRDX5 and PD-L1 is being carried on, which will be published in the near future. Furthermore, the identified residues of hPRDX5 are important to help in designing small peptides which can act as protein-protein interaction inhibitors with lower manufacturing costs and higher stability (Figure 7) for clinical therapeutic applications. This model provides the tool to fully use this unique immunotherapeutic pathway and rationally develop more effective and safer peptide inhibitors to block the protein-protein interactions involved in this pathway.

## MATERIALS AND METHODS

### Materials and reagents

*Escherichia coli* Trans 5α and BL21(DE3) strains and affinity chromatography ProBond resin (Ni^2+^) were bought from TransGen Biotech (Beijing, China) and Promega (Madison, WI), respectively. Trypsase, BamHI and HindIII were merchandised from New England Biolabs (Beijing, China). Tris base of molecular biology grade, NaCl, CaCl_2_, anhydrous Na_2_CO_3_, NH_4_HCO_3_, NaHCO_3_, and NaOH were purchased from Sigma-Aldrich Co. (St Louis, USA). HCl solution (37%), acetonitrile, formic acid (FA), glacial acetic acid were obtained from Thermo Fisher Scientific (Billerica, USA).

Ten percent sodium dodecyl sulfate (SDS) solution, ammonium persulfate, tricine, 5× sample buffer, glycine, 30% acrylamide/bis (29:1), and N,N,N',N' -tetramethylethylenediamine (TEMED) are electrophoresis purity reagents purchased from Bio-Rad Laboratories Inc. (Hercules, USA). Exact-Pro broad range (11-180kDa) pre-stained protein ladder was from Genstar (Beijing, China). Na_2_S_2_O_3_, Coomassie brilliant blue were purchased from Sigma-Aldrich Co. (St Louis, USA). Iso-propyl-thio-β-galactoside (IPTG), kanamycin and imidazole were purchased from Sigma (St Louis, USA). Bacto-yeast extract and Bacto-tryptone were obtained from OXOID (UK).

### Expression and purification of recombinant Human PRDX5

Human PRDX5 (GenBank Accession No. NM_012094), the short form without its mitochondrial pre-sequence, was expressed in *Escherichia coli* BL21(DE3) strain as 6×His-tagged fusion protein using pET28a(+) expression vector and purified as described previously (gPRDX5) [9]. Eventually, a pure protein of about 17 kDa was acquired by heterogenesis expression (Figure 1).

### Animal models and treatment

All animal experiments were conducted in accordance with The Standards for Laboratory Animals (GB14925-2001) and The Guideline on the Humane Treatment of Laboratory Animals (MOST 2006a) established by the People's Republic of China. And our protocols confirmed to the guidelines of Chinese Academy of Medical Sciences for experimental animal care and use.

### The model of C26-injected mice

Cachexia was induced by subcutaneous grafting of a 0.5 mm^3^ fragment of colon carcinoma (C26, obtained from the Institute of Basic Medical Sciences, Chinese Academy of Medical Sciences) in the dorsal region of 6- to 8-week-old BALB/c female mice. Mice were housed in standard conditions with day/night cycles of 12 hours and assigned to experimental and control groups (8 mice per group). Different concentrations of hPRDX5 (12.5, 25.0, 50.0, 75.0, 150.0 mg/kg) in sterile PBS (100 μl) were subcutaneously injected into the mice once a day for 20 days starting on day 5 prior to the inoculation of tumor. Control mice received the same volume of topotecan hydrochloride (TPT) (3.0 mg/kg), is of a wide range of applications in chemotherapy against various malignancies [39, 40], once three days after tumor transplantation (positive control) or PBS (solvent control) as described in hPRDX5.

### The model of melanoma B16-bearing mice

Experiments were performed using the model of melanoma B16 developed in C57Bl/6 male mice aged 6–8 weeks. The model of primary tumor growth (hereafter “subcutaneous melanoma”) was induced by subcutaneous inoculation of B16 cells (2 × 10^5^ cells per mouse, obtained from the Institute of Basic Medical Sciences, Chinese Academy of Medical Sciences) into the withers of each mouse. After tumor transplantation, the animals were assigned to experimental and control groups (8 mice per group) and were then kept in their compartments until the end of the experiment. The hPRDX5 of 75.0 mg/kg in sterile PBS (100 μl) was subcutaneously injected into the mice once a day for 20 days starting on day 5 prior to the inoculation of tumor. Control mice received the same volume of IL-2 (25.0 ng/kg) [41], IFN-γ (5.0 μg/kg) [42] once three days after tumor transplantation (positive control) or PBS (solvent control) as described in hPRDX5.

On 15th day after the tumor transplantation the mice were euthanized, tumor weight was measured, and the peripheral blood, thymuses and spleens were isolated for further analysis. The inhibition rate of tumor was calculated by the following equation: Inhibition rate (%) = (1-Tumor weight_test_/Tumor weight_solvent control_)×100%.

### Lymphocyte proliferation test

Cell proliferation was assessed as described by Swamy SM *et al* [43]. Splenocytes were obtained by gently placing the organ in RPMI-1640 medium (Sigma, USA) under aseptic conditions, followed by filtration and centrifugation (1000 rpm for 5 min) at room temperature. The erythrocytes were removed by hemolytic Gey's solution, while the remaining cells were centrifuged at 1000 rpm for 5 min. After two washes, the splenocytes were re-suspended in RMPI-1640 medium containing 10% fetal bovine serum (FBS), and adjusted to 1 × 10^7^ cell/ml. 100 μl of splenocyte suspension (1 × 10^7^ cell/ml) in a 96-well culture plate was cultured in RPMI-1640 medium containing 10% fetal bovine serum (FBS) with the addition of mitogens (LPS at 5, 10, 20 μg/ml or concanavalin A at 1, 5, 10, 20 μg/ml) (Sigma, USA). After incubation for 72 h at 37°C in a humidified 5% CO_2_ incubator, the number of proliferating cells was determined after centrifugation (1500 rpm for 10 min) by MTT assay [44] at a wavelength of 570 nm. The stimulation index was calculated by the following equation: Stimulation index = OD_test_/OD_control_.

### Determination of serum cytokine levels by the ELISA assay

The levels of IL-2, IL-4, IL-6, IL-10, TNF-α, TNF-β and IFN-γ in the serum of the mice from experimental and solvent control groups were determined using the mouse ELISA kits (Beyotime, China) according to the manufacturer's protocols.

### Molecular modeling

Discovery Studio 2.5 (Accelrys) was used for the modeling study and structure analysis. The representative crystal structures of hPRDX5 (1hd2) and PD-L1 (4z18) were obtained from the Protein Data Bank [45]. The potential interaction of hPRDX5 and PD-L1 was minimized and determined using the ZDOCK program [46, 47]. ZDOCK performs a fast Fourier transform search of all possible binding modes for proteins based on shape complementarity, desolvation energy, and electrostatics. Docking was carried out without specifying the binding residues so that the docking results will reflect the most possible interaction patterns without any arbitrary restrain. Of the 3600 poses generated, only the top 100 poses were retained. By manual analysis of the complexes, the important residues of Phe19, Asp122 and Tyr123 of PD-L1 involving in the interactions with PD-1 mentioned by Horita S *et al*. [37], were found in the complex that is ranked fifth on the basis of docking Z-Score.

### Statistical analysis

All statistical comparisons were carried out using one-way ANOVA test followed by Tukey's test (data are expressed as mean ± SD). *P*-values less than 0.05 were considered to be a statistically significant difference.

## CONCLUSIONS

In summary, the amino-acid sequence of gPRDX5, which is one of anti-cancer bioactive peptides (ACBPs), was identified in our lab. Meanwhile, we confirmed the anti-cancer bioactivity of gPRDX5 *in vitro* [9]. In this study, to overcome the immunogenicity between species for further therapeutic application, we got the sequence information of hPRDX5 by BLAST according to gPRDX5's sequence, and expressed the hPRDX5 protein with synthetic DNA sequence. Whereafter, the anti-cancer bioactivity of hPRDX5 was evaluated in colon cancer C26/BALB/c and melanoma B16/C57Bl/6 mice tumor models, and the results suggest that hPRDX5 could resist immunosuppression by promoting immune organs development, lymphocyte proliferation and up-regulation of the levels of serum cytokines. Moreover, the molecular docking study on hPRDX5/PD-L1 allows for a theoretical interpretation of PD-L1 immune blockade pathway by hPRDX5. In a word, our results provide a promising basis for getting more insights into PD-1/PD-L1's immune regulation and future development of the corresponding immunomodulating peptides related to hPRDX5 as drug candidates against corrcancers.
